# Epidemics in Partially Overlapped Multiplex Networks

**DOI:** 10.1371/journal.pone.0092200

**Published:** 2014-03-14

**Authors:** Camila Buono, Lucila G. Alvarez-Zuzek, Pablo A. Macri, Lidia A. Braunstein

**Affiliations:** 1 Instituto de Investigaciones Físicas de Mar del Plata, Departamento de Física, Facultad de Ciencias Exactas y Naturales, Universidad Nacional de Mar del Plata, Mar del Plata, Argentina; 2 Center for Polymer Studies, Boston University, Boston, Massachusetts, United States of America; Centre de Physique Théorique, France

## Abstract

Many real networks exhibit a layered structure in which links in each layer reflect the function of nodes on different environments. These multiple types of links are usually represented by a multiplex network in which each layer has a different topology. In real-world networks, however, not all nodes are present on every layer. To generate a more realistic scenario, we use a generalized multiplex network and assume that only a fraction 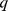 of the nodes are shared by the layers. We develop a theoretical framework for a branching process to describe the spread of an epidemic on these partially overlapped multiplex networks. This allows us to obtain the fraction of infected individuals as a function of the effective probability that the disease will be transmitted 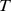. We also theoretically determine the dependence of the epidemic threshold on the fraction 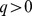 of shared nodes in a system composed of two layers. We find that in the limit of 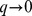 the threshold is dominated by the layer with the smaller isolated threshold. Although a system of two completely isolated networks is nearly indistinguishable from a system of two networks that share just a few nodes, we find that the presence of these few shared nodes causes the epidemic threshold of the isolated network with the lower propagating capacity to change discontinuously and to acquire the threshold of the other network.

## Introduction

Although the study of isolated networks allows us to understand how network topology affects network activity [1], most real-world networks are not isolated, instead they interact with other networks. In recent years, many researchers have studied how interconnections between networks produce phenomena that are absent in isolated networks [2]. A system composed of interconnected networks, often called a *network of networks* [3]–[6], retains connectivity links within each individual network but adds dependency links that connect each network to other networks in the system. This interdependency is the cause of many real-world multiple network phenomena, such as failure cascades [7], avalanches [8], and traffic overloads [9]. Very recently physicists have begun to consider a particular class of network of networks in which the nodes have multiple types of links across different *layers* [10]–[16]. These so-called multiplex networks were introduced in the social sciences several years ago [17] and provide a new way to advance the study of network complexity. They enable us to determine how the interplay between layers affects the dynamic processes running through them. This multiplex network approach has proven to be a successful tool in modeling a number of real-world systems, e.g., the European air transport system [18], [19] and the global cargo ship network [20].

The study of propagation processes in multiplex networks is a rapidly evolving research area. In particular, because of the urgent need for control strategies, the study of the propagation of disease epidemics has been the focus of much recent work. One of the most successful models used to describe the propagation of recurrent diseases is the susceptible-infected-susceptible (SIS) model. Research using the SIS model on multiplex networks [21]–[23] has found that the dynamics of the disease across a multiplex system is characterized by a critical point that is lower than the critical point of each isolated layer. Very recently Cozzo *et al.* [24] studied the SIS model in a multiplex network using a contact-contagion formulation with a rate of infection within each layer and a rate of infection between layers. They found that the critical point of the total system is always dominated by one of the layers. Although the SIS model can describe the propagation dynamics for recurrent diseases in which individuals are constantly being reinfected, there are many diseases in which ill individuals either die or after recovery become immune to future infections. For this class of disease, the favorite approach to describing the spreading process is the susceptible-infected-recovered (SIR) model [25]–[27]. At present there are only a few instances in which the SIR model has been applied to a network of networks. Dickison *et al.* [28] use the SIR model to numerically explore two interacting networks in order to determine the probabilities that the disease will spread within each individual network and between the networks of the system. Marceau *et al.* [29] developed an analytical approach that captures the dynamic interaction between two different SIR propagations over a multiplex network. Yagan *et al.* [30] studied the SIR model in a multiplex network with two different information layers, a *virtual* layer and a *physical* layer, each with different propagation speeds. They found that, even when the disease does not propagate in a particular layer, an epidemic can occur in the conjoint virtual-physical network.

In social interactions, individuals are not necessarily present in all layers of a society. To allow for this significant constraint, we use a *partially overlapped multiplex* network in which only a fraction of individuals are present in all layers. Our goal is to study how this overlapping fraction affects the spreading of such nonrecurrent diseases as influenza, the H5N5 flu or the Severe Acute Respiratory Syndrome (SARS) [31]. We use the SIR model over a partially overlapped multiplex network. In the SIR model each individual of the population can be in one of three different states: susceptible, infected, or recovered. Infected individuals transmit the disease to its susceptible neighbors with a probability 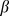 and recover after a fixed time 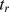. The spreading process stops when all the infected individuals are recovered. The dynamic of the epidemic is controlled by the transmissibility 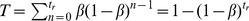, which is a measure of disease virulence, i.e., the effective probability that the disease will be transmitted across any given link. As in the SIR model, an individual cannot be reinfected, the disease spreads through branches of infection that have a tree-like structure, and thus can be described using a generating function formalism [32], [33] that holds in the thermodynamic limit.

We first examine some of the concepts of the generating function formalism for an isolated network, and we then extend this formalism to the partially overlapped multiplex network. In the generating function framework, the relevant magnitude that provides information about the process is the probability 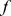 that a branch of infection can extend throughout the network [34], [35]. When a branch of infection reaches a node of connectivity 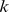 across one of its links, the branch can only expand through its remaining 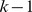 connections. Thus the probability that a node of connectivity 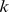 belongs to a branch of infection is proportional to 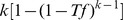, since the probability to reach a node through a link is proportional to its connectivity. Thus 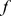 verifies the self-consistent equation 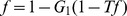 in isolated networks, where 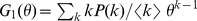 is the generating function of the underlying branching process [33], 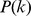 is the degree distribution, and 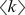 is the average degree of the network. In the steady state of the epidemics, the branches of infection form a single cluster of recovered individuals made up of nodes that were infected by some of its connections. Thus the fraction of nodes in the cluster of infection of an isolated network is given by 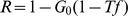, where 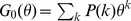 is the generating function of the degree distribution. Within this formalism we find that the self-consistent equation has a nontrivial solution above the critical transmissibility 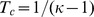, where 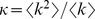 is the branching factor and 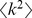 is the second moment of 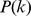. Since 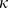 can be used to measure the connectivity dispersion of the network, we find that the critical threshold is very small for heterogeneous networks. At this critical threshold, the fraction of recovered individuals 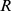 overcomes a second-order phase transition where at 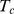 and below 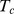 the disease cannot spread and above 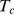 the disease infects a significant fraction of the population and becomes an epidemic. Therefore an epidemic occurs only if the number of recovered individuals in the steady state reaches or exceed a minimum size 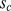. In this letter, we use 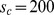 for all our simulations [36].

## Method

**Figure 1:**
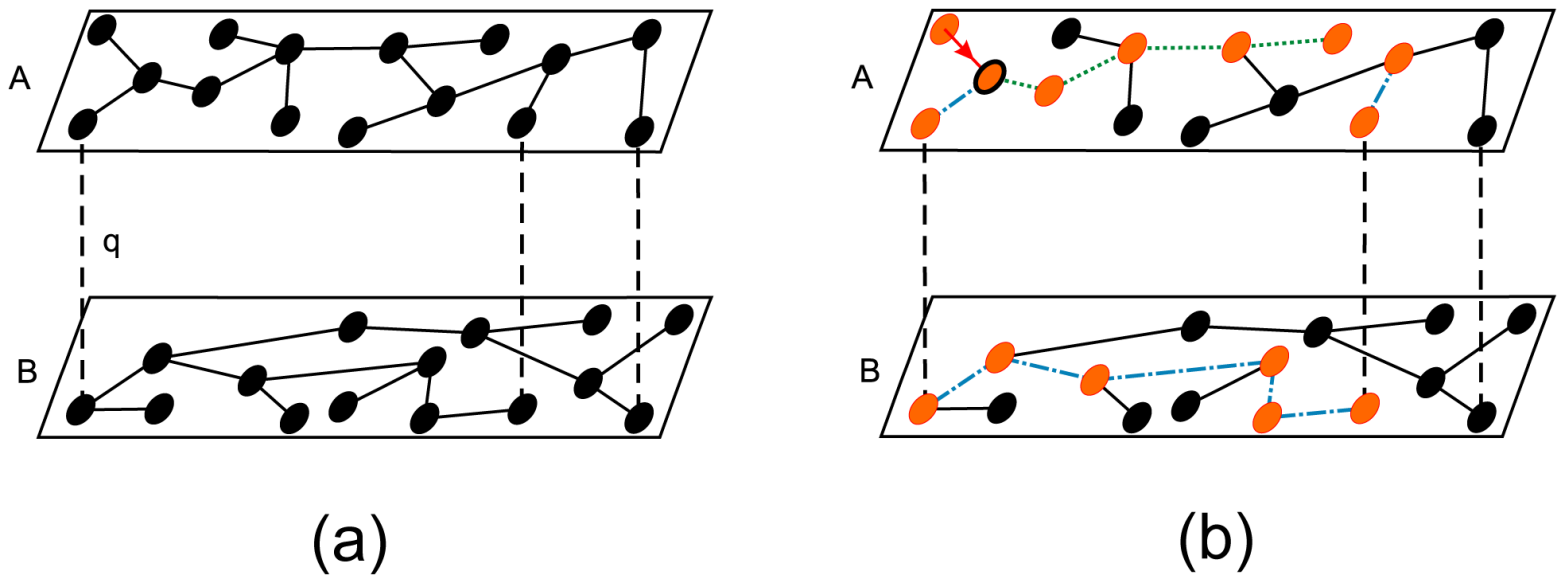
Scheme of a SIR epidemic process in a partially overlapped multiplex network. Partially overlapped multiplex network with layer size 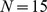 and fraction of shared nodes 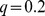. The total size of the network is 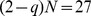 individuals. The dashed lines are used as a guide to show the fraction 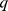 of shared nodes. (a) Before the spreading dynamics, all individuals are in the susceptible stage represented by black circles. (b) In the steady state of the epidemic, the recovered individuals are denoted by orange circles. The branches of infection start in the link denoted by a red arrow, which leads to an infected orange node denoted with a black contour. Two branches expand through the two available links of that node. One of the branches denoted by green dotted lines corresponds to a branch of infection that only spreads through layer A that is described by the first term of 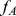 in Eq. (1). The other branch denoted in blue dash-dotted lines is a branch of infection that spreads through both layers and is described by the second term of 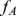 in Eq. (1). An analogous interpretation holds for the terms of 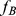 of Eq. (1).

In our model we use an overlapping multiplex network formed by two layers, A and B, of the same size 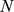, where an overlapping fraction 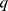 of *shared* individuals is active in both layers. Figure 1(a) shows schematically the partially overlapped network. The dashed lines that represent the fraction 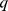 of shared individuals should not to be interpreted as interacting or interdependent links but as the shared nodes and their counterpart in the other layer.

For the simulation, we construct each layer using the Molloy Reed algorithm [37], we choose randomly a fraction 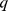 of nodes in each of the layers that represent the same nodes. In our model of the SIR process we assume that the transmissibility is the same in both layers because there is only one disease and all individuals in the system spread equally. We begin by infecting a randomly chosen individual in layer A. The spreading process then follows the SIR dynamics in both layers, the overlapped nodes in both layers have the same state because they represent the same individuals. After all infected nodes infect their susceptible neighbors with probability 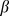 in both layers, the time is increased in one, and the states of the nodes are updated simultaneously. Note that because there are shared nodes the branches of infection can cross between the two layers. Thus the probability that, following a random link, a node belonging to the infected cluster will be reached in each layer can be written



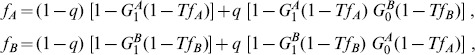
(1)where 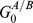 and 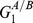 are the generating functions defined above for layer A and B, respectively. In Eq. (1) both 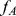 and 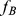 are written as the sum of two terms that takes into account all possible spreading of the branches of infection. The first term corresponds to those branches of infection that only spread within their own layer, while the second term takes into account those branches that spread through both layers. Figure 1(b) shows how a node is reached through an ingoing link marked by an arrow. The disease spreads through both available outgoing links of that node in layer A and develops two branches of infection. The green dotted line denotes the branch that stays in layer A and corresponds to the first term of Eq. (1) for 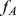. The second term of Eq. (1) for 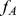 is indicated by the blue dot-dashed branch that reaches layer B through a shared node and then spreads to its neighbors on that layer. After the shared node is infected, the branch spreads through five links in layer B and reaches another shared node that allows the branch of infection to spread back to layer A. An analogous interpretation holds for the terms of 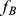.

## Results

**Figure 2:**
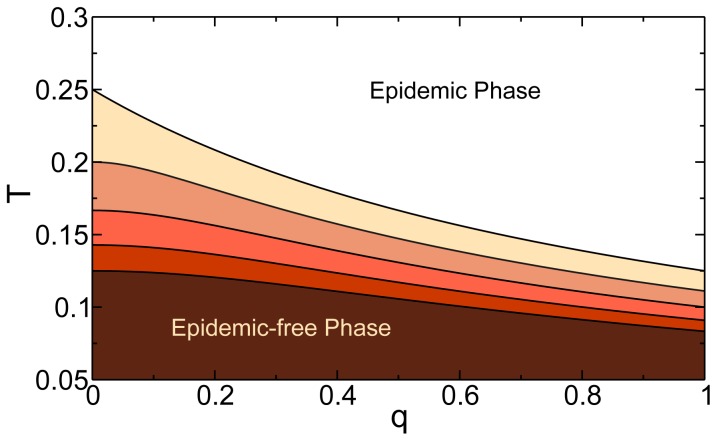
Dependence of the epidemic threshold of the SIR model with the overlapping fraction and topology of the layers. Phase diagram in the 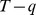 plane for two Erdös-Rényi layers with 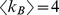 and different values of 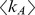. The black full lines correspond to 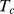 obtained theoretically from Eq. (3) for 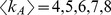 from top to bottom. The limit 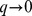 corresponds to a disease spreading in layer A when it is isolated and the limit 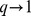 represents the fully overlapped multiplex network. Colored regions correspond to the epidemic-free phase for each value of 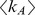, while the region above 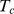 corresponds to the epidemic-phase.

The solution of Eq. (1) for all 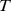 above and at criticality is given by the intersection of 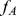 and 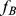, which can be derived by solving the determinant equation 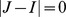, where 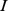 is the identity and 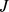 is the Jacobian matrix of Eq. (1). The only possibility to have a non-epidemic regime is that none of the branches of infection spread, *i.e.* 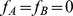. Therefore below and at criticality 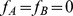, an evaluation of the Jacobian matrix 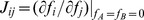 given by
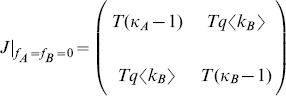
(2)allow us to obtain a quadratic equation for 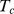 with only one stable solution [38] given by,
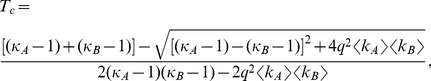
(3)where 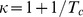 is the total branching factor and 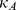, 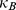 are the isolated branching factors of layer A and B respectively. For 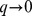 we recover the isolated network result 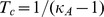, which is compatible with our model in which the infection starts in layer A and, because 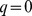, the disease never reaches layer B. In contrast, when 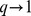, we find 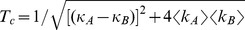. Note that 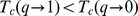. In general, 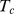 decreases as a function of 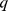. This is the case because an increase in the overlapping between layers causes an increasing in the dispersion of the degrees of the nodes, therefore the total system becomes more heterogeneous in degree making the total branching factor to increase, *i.e.*, the total branching factor is equal to or bigger than the branching factor of the isolated layers. Figure 2 shows this behavior with a plot of a phase diagram in the plane 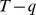 for Erdös-Rényi (ER) layers [39] whose degree distribution is Poissonian 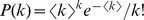 and its branching factor is given by 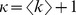. Figure 2 shows the critical lines 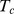 given by Eq. (3) as a function of the overlapping fraction 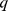 when one of the layers is fixed at 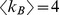 for the different average connectivities 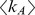 of layer A. The colored areas correspond to the epidemic-free phase for a given connectivity in layer A, and the region above the critical lines belongs to the epidemic phase. The left and right extremes of the critical lines correspond to the limits 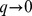 and 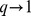 for Eq. (3) mentioned above.

**Figure 3:**
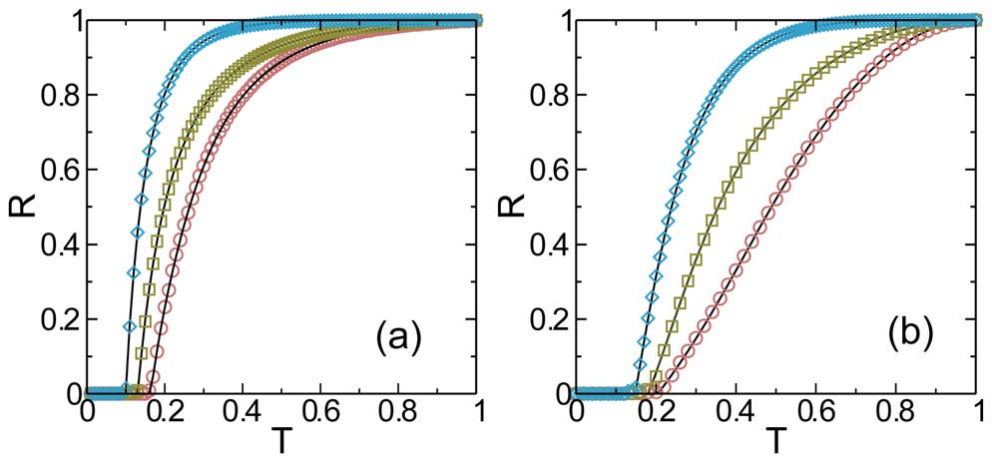
Theoretical predictions and simulations for the fraction of recovered individuals in the steady state of the epidemics. Total fraction of recovered individuals in the steady state of the SIR model with 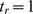 for (a) two Erdös-Rényi layers with 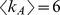 and 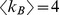 and for (b) two power law layers with exponential cutoff 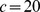 with 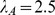 and 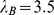, the minimum and maximum values of 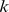 where set as 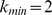 and 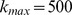, respectively, for both layers. In both panels full black lines correspond to theory given by Eq. (5) and simulations results are given for 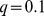 in pink circles, 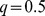 in green squares and 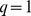 in blue diamonds. All simulations were done with a total system size of 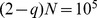 and over 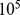 realizations.

In the steady state, the fraction of nodes reached by the branches of infection, i.e., the recovered individuals in each layer, can be written



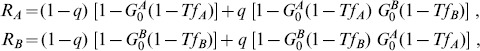
(4)and the total fraction of recovered individuals 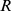 is given by
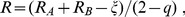
(5)where 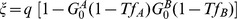 is the fraction of shared nodes that have recovered in the steady state. The factor 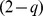 appears because the total number of individuals in the system is 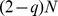. Figure 3 plots the total fraction 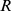 of recovered individuals, obtained from Eq. (5), as a function of 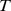 for different values of the overlapping fraction 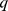 and compares it with simulation results for 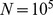 nodes and 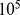 realizations. Figure 3 shows the results for (a) two ER layers with 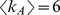 and 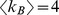 and (b) two power law distributed layers with an exponential cutoff 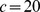, where 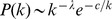, and exponents 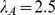 and 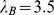. In both cases we observe the typical second order phase transition of the SIR process with the transmissibility 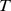 as the control parameter—with perfect agreement between the theory and the simulations. As the overlapping fraction 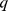 increases [see Eq. (3)] the critical threshold moves to the left and the increase in 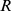 becomes more abrupt but the second-order character of the SIR for isolated networks is preserved [40]. In the case of the power-law distributed layers, when 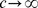, 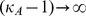, which eliminates any dependence of the critical threshold on 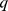, as can be inferred from Eq. (3).

**Figure 4:**
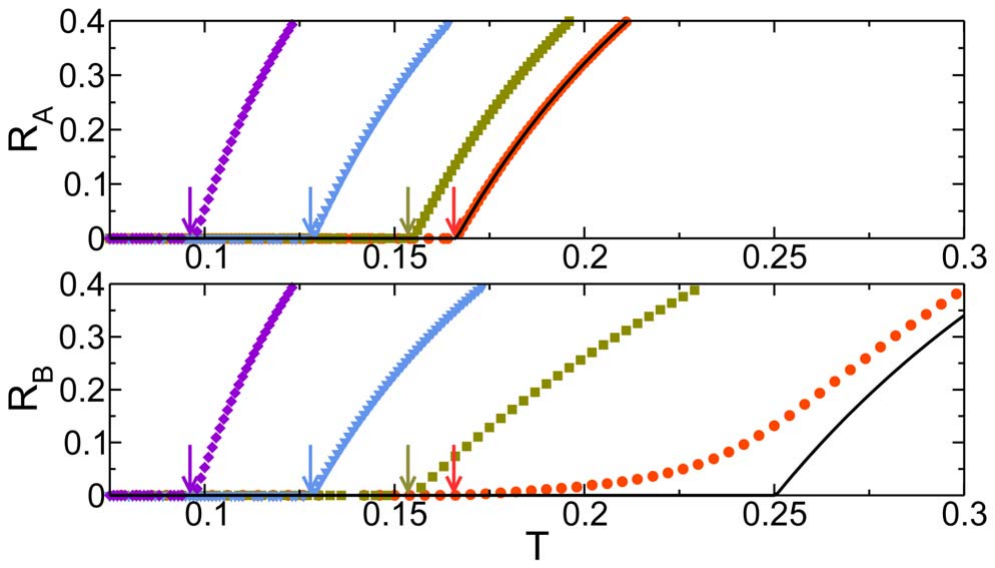
Effect of the overlapping fraction in the SIR epidemic threshold on individual layers. Fraction of recovered individuals vs the transmissibility in the steady state of the SIR model. The values were obtained theoretically from Eq. (4) for two Erdös-Rényi layers with 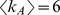, 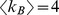 and different overlapping fraction values. In orange circles 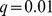, in green squares 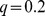, in blue triangles 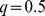 and in violet diamonds 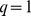. In the upper panel we plot 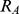 and in the bottom panel we plot 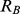. The arrows indicate the threshold 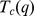 and are used as a guide to show that 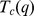 is the same for 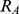 and 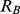. The black full lines denote 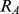 (up) and 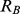 (down) when both networks are isolated and 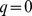.

Finally we investigate the effect of the overlapping fraction by observing the epidemic in each layer separately, shown in Fig. 4. When 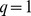, the threshold [see Eq. (3)] is at its minimum and both layers have the same fraction of recovered nodes. This is the case because the layer with the bigger isolated threshold (or the smaller isolated branching factor) can be infected by either its own infection branches or by those coming from the other layer. This second possibility decreases with 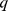. For lower values of 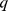 the epidemic threshold increases because the total branching factor is lower and the layer with the lower isolated threshold cannot as effectively infect the other layer. As a consequence, when 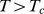 the fraction of recovered individuals of the layers detach from each other and show a difference that increases as 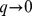 [see Eq. (3)]. In this limit, the joined threshold approaches quadratically the threshold of the isolated layer with the higher branching factor. Thus no matter how small the overlapping fraction is, when 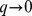 the epidemic threshold of the system is given by the lower isolated threshold that corresponds to the layer with the higher propagation capability. This limit is consistent with the results found in Ref. [24] for the SIS model in which the epidemic threshold of the system is dominated by the layer with the lower isolated threshold. Thus although a system of two completely isolated layers is indistinguishable from a system of two layers that share only a few nodes (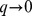), the isolated epidemic threshold of the less propagating layer will change discontinuously and acquire the isolated threshold of the other layer.

## Discussion

In summary, we have studied a SIR epidemic propagation model in a partially overlapped multiplex network formed by two layers that share a fraction 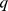 of nodes. We find that the epidemic threshold 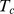 of the multiplex network depends on both the topology of each layer and the overlapping fraction 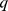. Using of a generating function framework, we find the equation for the threshold 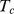 and also the equation for the recovered individuals in the steady state of the spreading process. Our analytical predictions are in agreement with extensive simulation results. Finally, we analyze the fraction of recovered individuals in the steady state as a function of the transmissibility 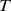 for layer A and layer B separately. When 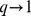, we find that the epidemic threshold is at its minimum and, because all individuals belong to both layers, that both layers have the same fraction of recovered nodes for all 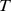. As 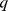 decreases, the total branching factor of the system decreases and the epidemic threshold increases, and when 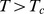 the fraction of recovered individuals in both layers detach from each other. When 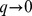, the epidemic threshold of the system is dominated by the isolated epidemic threshold of the layer with the larger propagation capability and thus it reaches a higher value. Thus although a system of two completely isolated layers is indistinguishable from a system of two layers that share only a few nodes, the presence of these few shared nodes causes the epidemic threshold of the isolated network with the lower propagating capability to discontinuously change to the threshold of the other network. This result may have important implications for the implementation of non-pharmaceutical interventions to control the propagation of diseases on real scenarios. Our study suggests that vaccinating or isolating only that layer with the higher propagation capacity can drastically reduce the total branching factor of the network, as can be seen from Eq. (3). As a consequence, the epidemic threshold of the system increases significantly, and the risk that a disease epidemic will propagate across the entire network is reduced.
