# Correction: Epidemics in Partially Overlapped Multiplex Networks

**DOI:** 10.1371/journal.pone.0104373

**Published:** 2014-07-29

**Authors:** The PLOS ONE Staff

There are errors in two equations in the published article. In the Results section, Equation (2) is represented incorrectly. The correct equation, provided by the authors, can be viewed here: 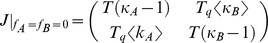



There is an error in the fourth sentence of the second paragraph of the Results section. The correct sentence should read: Figure 3 shows the results for (a) two ER layers with 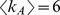 and 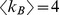 and (b) two power law distributed layers with an exponential cutoff 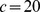, where 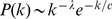, and exponents 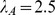 and 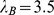.

## References

[1] Buono C, Alvarez-Zuzek LG, Macri PA, Braunstein LA (2014) Epidemics in Partially Overlapped Multiplex Networks. PLoS ONE 9(3): e92200 doi:10.1371/journal.pone.0092200 24632709 10.1371/journal.pone.0092200 PMC3954885

